# Determining the interlayer shearing in twisted bilayer MoS_2_ by nanoindentation

**DOI:** 10.1038/s41467-022-31685-7

**Published:** 2022-07-06

**Authors:** Yufei Sun, Yujia Wang, Enze Wang, Bolun Wang, Hengyi Zhao, Yongpan Zeng, Qinghua Zhang, Yonghuang Wu, Lin Gu, Xiaoyan Li, Kai Liu

**Affiliations:** 1 State Key Laboratory of New Ceramics and Fine Processing & Key Laboratory of Advanced Materials of Ministry of Education, School of Materials Science and Engineering, Tsinghua University, Beijing, 100084 China; 2 Center for Advanced Mechanics and Materials, Applied Mechanics Laboratory, Department of Engineering Mechanics, Tsinghua University, Beijing, 100084 China; 3 Institute of Physics, Chinese Academy of Sciences, Beijing, 100190 China

**Keywords:** Structural properties, Two-dimensional materials

## Abstract

The rise of twistronics has increased the attention of the community to the twist-angle-dependent properties of two-dimensional van der Waals integrated architectures. Clarification of the relationship between twist angles and interlayer mechanical interactions is important in benefiting the design of two-dimensional twisted structures. However, current mechanical methods have critical limitations in quantitatively probing the twist-angle dependence of two-dimensional interlayer interactions in monolayer limits. Here we report a nanoindentation-based technique and a shearing-boundary model to determine the interlayer mechanical interactions of twisted bilayer MoS_2_. Both in-plane elastic moduli and interlayer shear stress are found to be independent of the twist angle, which is attributed to the long-range interaction of intermolecular van der Waals forces that homogenously spread over the interfaces of MoS_2_. Our work provides a universal approach to determining the interlayer shear stress and deepens the understanding of twist-angle-dependent behaviours of two-dimensional layered materials.

## Abstract (web summary)

The study of the mechanical properties of twisted van der Waals structures can provide important information about their interlayer coupling and electronic behaviour. Here, the authors report a nanoindentation-based technique to determine the interlayer shear stress in bilayer MoS2, showing its independence as a function of the twist angle.

## Introduction

Two-dimensional (2D) layered materials have attracted considerable attention in the past decade owing to their physical and chemical properties. Their atomically flat, dangling-bond free surface enables van der Waals (vdW) stacking and the integration of 2D materials into 3D architectures, providing an additional dimension for the modulation of material properties^1–3^. The twist angle, which determines the vdW stacking direction from one layer of 2D material to another one, should influence the properties of integrated 2D materials^4–7^ but was commonly ignored in the early studies of 2D electronic devices. However, a recent study has shown that a vdW-stacked bilayer graphene exhibits superconductivity at a specific twist angle of 1.1° (the first “magic” angle)^8^, leading to the rise of “twistronics”. In recent years, twist-angle-dependent correlated insulator states, Moiré excitons, stacking-dependent interlayer magnetism, and topological polaritons have been discovered^9–13^. Inspired by these studies, there are growing demands to understand in depth how the twist angle influences the interlayer coupling of 2D homo- or heterostructures. Although many studies have focused on electronic interlayer coupling in twistronics, the relationship between interlayer mechanical interactions and twist angles has yet to be reported.

It is of importance to clarify the relationship between the twist angle and the interlayer mechanical interaction in vdW-integrated architectures, which, in particular, benefits the design of 2D flexible electronics^14^. In 2D layered systems, the overall robustness is determined by the interlayer mechanical interaction rather than the mechanical strength of each individual layer, as the interlayer vdW forces are much weaker than the intralayer chemical bonding forces^15^. Unfortunately, current methods of measuring interlayer interactions of 2D materials, including the pressurized bubbling method^16–19^, tip-based adhesion force measurement^20–22^, and nanoindentation^23–28^, have certain limitations when probing the twist-angle-dependent interlayer interaction of 2D materials in monolayer limits. For instance, pressurized bubbling tests require the ultimate gas impermeability of detected materials to determine their interlayer shear stresses, and thus, the detected materials are usually limited to graphene^16–18^. The tip-based adhesion force measurement could determine the adhesive force between the 2D material-wrapped tip and the target 2D material^20,21^, yet this method does not have twist-angle-resolved capability. Nanoindentation has been widely used to measure the elastic moduli of 2D materials by indenting suspended regions of 2D materials. It could also qualitatively probe the interlayer interactions of bilayer or multilayer 2D materials because weaker interlayer interactions induce greater attenuation of the effective elastic moduli, which are lower than the overall moduli counting each layer^23–28^. However, it is still challenging to quantitatively determine the interlayer shear stress because the indentation induces tensile stress and shear stress simultaneously at the suspended region of bilayer or multilayer 2D materials.

**Fig. 1:**
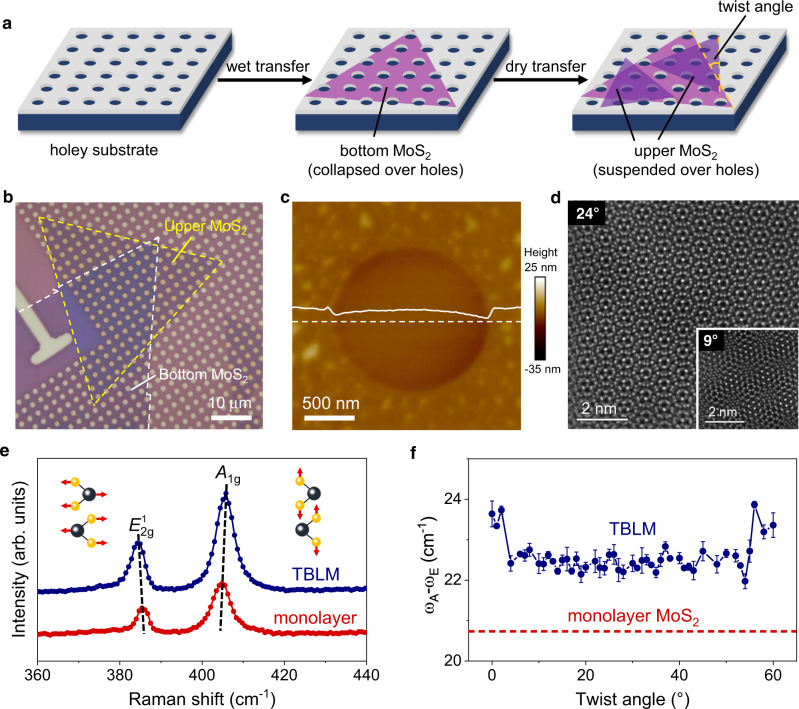
Preparation and characterization of twisted bilayer MoS_2_ (TBLM). **a** Schematic diagram of the preparation of TBLM. Here, the bottom layer MoS_2_ collapses over the holes, while the upper layer is suspended over the holes. **b** Optical image of a TBLM sample. The dashed lines represent the edges of upper and bottom MoS_2_ monolayers. **c** Atomic force microscope surface topology image of TBLM over a single hole. The white solid line is the height profile of the sample across the white dashed line. The variation of the height profile is 4.1 nm. **d** Clear Moiré patterns of TBLM at twist angles of 24° and 9° (inset) observed under annular dark-field scanning transmission electron microscopy. **e** Comparison of the Raman spectra of TBLM and monolayer MoS_2_. The increase in peak interval is illustrated by the two black dashed lines. The inset shows the vibrational modes of ${E}_{2{{{{{\rm{g}}}}}}}^{1}$ and *A*_1g_. **f** Twist-angle dependence of the peak interval in TBLM samples on a SiO_2_/Si substrate. The red dashed line indicates the peak interval between ${E}_{2{{{{{\rm{g}}}}}}}^{1}$ and *A*_1g_ of monolayer MoS_2_.

In this work, we established an experimental configuration together with theoretical model to probe the twist-angle-dependent interlayer interaction of twisted bilayer MoS_2_ (TBLM) by nanoindentation. Experimentally, this is realized by first selectively breaking the suspending region of the bottom layer of TBLM over circular holes on a substrate and then twistedly stacking the upper layer onto the bottom layer and keeping the upper layer intactly suspended over the holes (Fig. 1a). In this configuration, the suspended region of the TBLM is only from the upper layer, which is constrained by the bottom layer around the edges of the holes. As a result, the tensile and shear regions of the upper layer MoS_2_ are separated, and the shearing/sliding interaction between the two layers only occurs at the boundaries around the edges of the holes. This experimental configuration enables us to build a clear realistic theoretical model based on shearing boundaries to describe the interlayer interaction of the TBLM. Although the nonplanar crystal structure of MoS_2_ (i.e., one layer of Mo atoms sandwiched by two layers of S atoms) implies a significant steric effect^29^ and thus a twist-angle-dependent interlayer mechanical interaction, our results show that the interlayer shearing interaction is surprisingly independent of the twist angle. With the shearing-boundary mechanical model, the average interlayer shear stress of the TBLM is quantitatively determined to be ~2.51 MPa. This value is much lower than that of the MoS_2_@SiO_2_ interface (11.09 MPa), suggesting that TBLM is more prone to interlayer shearing than monolayer MoS_2_ laid on SiO_2_. Molecular dynamics (MD) simulations further confirm the twist-angle-independent interlayer mechanical interaction, and the derived theoretical interlayer shear stress is very consistent with our experimental data. The independence of the interlayer shear stress of TBLM is attributed to the fact that the overall interlayer vdW force is the sum of intermolecular forces, which homogenously spread over the TBLM interfaces. Our work provides a universal approach to quantitatively evaluate the interlayer interactions of various 2D materials and their heterostructures. The twist-angle-independent shear stress also sheds light on the fabrication and application of 2D vertical heterostructures.

## Results and discussion

### Preparation and characterization of TBLM

High-quality MoS_2_ monolayers were grown on SiO_2_/Si substrates (Supplementary Fig. 1) under ambient pressure using MoO_3_ and sulfur as precursors with the assistance of perylene-3,4,9,10-tetracarboxylic acid tetrapotassium salt (PTAS), which is similar to previous reports^30,31^. To carry out the nanoindentation tests, a SiO_2_/Si substrate was prepatterned with arrays of circular holes with a depth of 300 nm and a diameter of either ~1.0 μm or ~1.5 μm^32^. TBLM was prepared by two-step transfer processes that include a polymethyl methacrylate (PMMA)-assisted wet transfer to break the bottom MoS_2_ monolayer region over holes, followed by a polydimethylsiloxane (PDMS)-assisted dry transfer to stack and suspend the upper MoS_2_ monolayer region over holes (Fig. 1a, also see Methods for details). After the wet transfer, the bottom MoS_2_ monolayers collapsed over holes and exhibited sharp edges around the holes, while only the supported region remained on the substrate (Supplementary Fig. 2). Then, after the dry transfer, the upper MoS_2_ monolayers were randomly stacked onto the bottom monolayers, forming TBLM with random twist angles (Fig. 1b). No solvent treatment was involved when the PDMS was peeled off in the dry transfer to keep the suspended region of the upper monolayers intact. Either the wet or dry transfer process was kept clean in all aspects to guarantee clean surfaces of MoS_2_ monolayers (Supplementary Fig. 1). The sample was also annealed after either transfer process to remove any polymer residues and have the twisted bilayer interact effectively. This random stacking is efficient for preparing clean TBLM with various twist angles to obtain abundant angle-resolved data. As the MoS_2_ monolayers exhibit regularly triangular shapes, the twist angle of TBLM can be directly determined by identifying the crystal orientations of the upper and bottom MoS_2_ monolayers under an optical microscope (Fig. 1b). After the two-step transfer, only the upper MoS_2_ monolayers are suspended over the holes and constrained by the bottom MoS_2_ monolayers around the edges of the holes (Fig. 1c). There only exists in the TBLM samples a very limited density of bubbles or wrinkles with a coverage reaching the lows in the twisted samples reported (Supplementary Fig. 3). Furthermore, clear Moiré patterns observed under annular dark-field scanning transmission electron microscopy (ADF-STEM) also suggest the high-quality and clean interfaces of the TBLM samples (Fig. 1d).

Raman spectroscopy can be used to probe the interlayer coupling of MoS_2_ with the fingerprint out-of-plane and in-plane vibrational modes, namely, *A*_1g_ and ${E}_{2{{{{{\rm{g}}}}}}}^{1}$, respectively. The peak interval between *A*_1g_ and ${E}_{2{{{{{\rm{g}}}}}}}^{1}$ has been found to be sensitive to the number of layers and the twist angle of MoS_2_ owing to the interlayer coupling and different symmetries^33^. Figure 1e shows that the interval between these two characteristic Raman peaks of MoS_2_ increases by ~2 cm^−1^ for TBLM compared to monolayer MoS_2_, indicating the existence of a strong interlayer coupling of TBLM^33,34^. Figure 1f and Supplementary Fig. 4 show that the peak interval is largest (~23.5 cm^−1^) when the twist angle is close to or equal to 0 and 60°, while it remains a constant value of ~22.5 cm^−1^ for other twist angles. These results correspond well with a previous study on TBLM directly grown by CVD^4^, indicating that our transfer method works for the preparation of TBLM with strong interlayer coupling.

### Nanoindentation experiments of TBLM

**Fig. 2:**
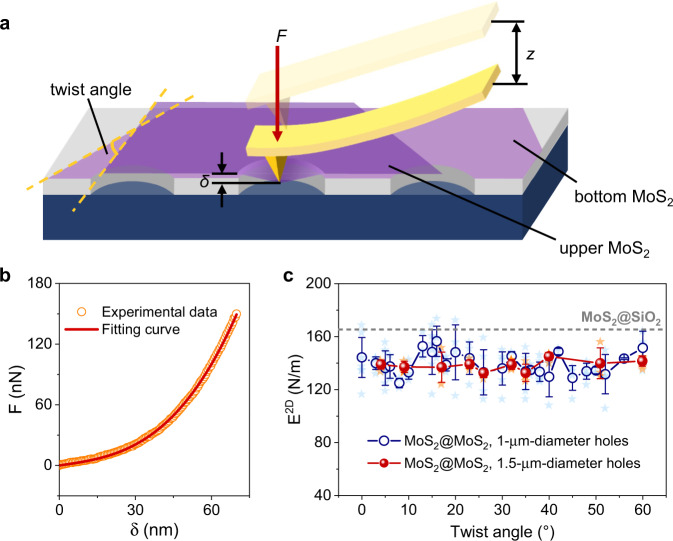
Nanoindentation experiments of TBLM. **a** Schematic diagram of nanoindentation tests. *F* is the point force applied to the suspended region of the sample, *z* is the moving distance between the tip and the sample, and *δ* is the indentation depth of the suspended membrane. **b** Typical *F*-*δ* curve (orange circles) of a TBLM sample with the red line showing a fitting of the *F*-*δ* curve following Eq. (1). **c** Dependence of measured in-plane elastic moduli of TBLM on twist angles. Blue stars and orange stars mark the measured moduli of each suspended membrane over 1-μm-diameter and 1.5-μm-diameter holes, respectively. The blue circles and red spheres are the corresponding average values. Error bars represent standard deviations. The gray dashed line represents the in-plane moduli of monolayer MoS_2_ on SiO_2_.

We conducted nanoindentation experiments under an atomic force microscope (AFM) by applying a point force *F* to the suspended region of a sample (Fig. 2a). The force can be calculated as *F* = *kx*, where *k* and *x* are the spring constant and the displacement of the AFM probe, respectively. Here, *k* is calibrated by the Sader method (online calibration), which follows a simple harmonic oscillation model^32^, and *x* is given by the AFM system. The indentation depth *δ* of the suspended membrane can be derived as *δ* = *z*-*x*, where *z* is the moving distance between the tip and the sample, as illustrated in Fig. 2a. Previous theoretical studies based on the fixed-boundary model, which means the suspended membrane is firmly clamped at the edge of the hole during nanoindentation, gave the following *F*-*δ* expression for nanoindentation of thin membranes^35–37^:

1 $$F\,=({{\sigma }_{0}}^{2{{\mbox{D}}}}\pi )\delta \,+\,({E}^{2{{\mbox{D}}}}\frac{{q}^{3}}{{a}^{2}}){\delta }^{3}$$

where *E*^2D^ is the in-plane elastic modulus in units of N/m, *σ*_0_^2D^ is the pretension of the suspended membrane in units of N/m, *a* is the radius of the hole, and *q* = 1/(1.05–0.15*ν*–0.16*ν*^2^) is a factor determined by Poisson’s ratio *ν*. For MoS_2_, we take *ν* *=* 0.27 and *q* = 1.00, following previous studies^26,27^. Figure 2b shows a typical *F*-*δ* curve. By fitting the *F*-*δ* curve using Eq. (1), we can obtain *σ*_0_^2D^ and *E*^2D^, as shown by the red line in Fig. 2b (the data processing can be seen in Supplementary Fig. 5). Equation (1) includes the asymptotic solutions at small and large displacements. For the small displacement, the one-order term related to pretension is dominant. For the large displacement, the cubic term related to the in-plane modulus is dominant. Because Eq. (1) captures the main deformation features (especially the cubic term at the large displacement) of nanoindentation and has a simple and explicit expression, it has been widely used to extract the in-plane stiffness (or in-plane modulus) of various 2D materials from nanoindentation force–displacement curves^23,25,26,28,37^. Note that Eq. (1) does not consider the influence of the indenter tip radius, and thus it introduces a certain error^38^. However, the accuracy of extracting the in-plane modulus from the nanoindentation force–displacement curve is mainly determined by the cubic term in Eq. (1). If only there are enough experimental data falling in the large displacement regime (i.e., following the cubic term), it is possible to use Eq. (1) to determine the modulus with high precision^28^.

We tested 113 TBLM samples with 26 twist angles over 1-μm-diameter holes and 46 TBLM samples with 10 twist angles over 1.5-μm-diameter holes. On each sample, we performed 3–5 consecutive nanoindentations under different loads, typically ranging from 90 to 450 nN, to measure the pretensions and moduli of TBLM samples. The corresponding indentation depth is much smaller than the diameter of holes (30–70 nm for holes in 1 μm diameter and 60–130 nm for holes in 1.5 μm diameter) under these moderate loads, and as a result, the strain applied on the upper MoS_2_ monolayer is estimated to be less than 2% for all of the nanoindentation measurements. Under such small strains, both the deformation of the suspended upper monolayer region and the shearing at the twisted bilayer region are elastic rather than plastic, and the in-plane deformation of the upper monolayer is very minor compared with the twist-angle-induced lattice mismatch. The *F*-*δ* curves in five consecutive nanoindentations under different loads follow nearly identical traces until the breaking of the upper MoS_2_ monolayers (Supplementary Fig. 6), and the measured *E*^2D^ does not change with time (Supplementary Fig. 7,  *E*^2D^ varies <5% in 2 months), suggesting very good reproducibility of our measurements. The unchanged surface topology of a TBLM sample reveals no wrinkling before and after nanoindentation (Supplementary Fig. 8). This fact excludes the wrinkling effect^39^ that may be induced by nanoindentation and simplifies our model, as will be discussed later.

By fitting the force curves with Eq. (1), we obtained the *E*^2D^ of each nanoindentation and averaged the values for each sample. Figure 2c shows the dependence of *E*^2D^ on the twist angles. The data dispersion originates from many aspects in our experiments, such as the difference between single-crystal flakes, the offset of indentation positions, and the deviation of measured hole sizes. Note that the data obtained from the samples over larger holes (~1.5 μm in diameter) have smaller deviations. Considering these deviations in the force measurements, the in-plane moduli of MoS_2_ seem to remain relatively constant regardless of the twist angles (also see Supplementary Fig. 9), either for the samples over 1-μm-diameter holes or for those over 1.5-μm-diameter holes. This is a surprising result because for a MoS_2_ monolayer, Mo atoms are sandwiched between two layers of S atoms, forming a nonplanar structure^29^, which implies that MoS_2_ should exhibit a significant steric effect and that the interlayer mechanical interaction is likely to depend on the twist angle.

There are two contradictory hypotheses that can be put forward to explain our experimental results. One is that the interlayer interaction may be dependent on the twist angle, but the interaction is strong enough to have the upper MoS_2_ monolayer fulfill the fixed-boundary condition, and thus all the measured moduli should be equal to the intrinsic value of MoS_2_ regardless of the twist angle. The other hypothesis is that the weak interlayer mechanical interaction has already softened the upper MoS_2_ monolayer, but it is independent of the twist angle, so its impact on the measured moduli is identical. To clarify this, the boundary conditions of the suspended upper monolayer must be examined in depth.

### Investigation of the fixed-boundary condition model

**Fig. 3:**
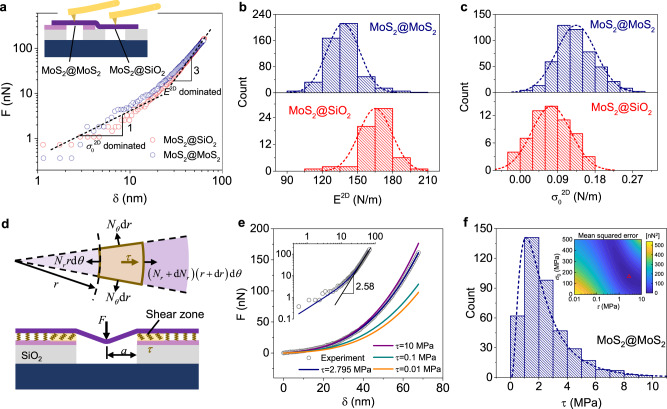
Determination of shear stress at TBLM interfaces with the shearing-boundary model. **a** Typical force-indentation depth (*F*-*δ*) curves of MoS_2_@MoS_2_ (blue dots) and MoS_2_@SiO_2_ (red dots) in logarithmic coordinates. The inset shows a schematic diagram of MoS_2_@MoS_2_ and MoS_2_@SiO_2_. **b**, **c** Histograms of the in-plane elastic modulus (*E*^2D^) and pretension (*σ*_0_^2D^) of all samples measured over 1-μm-diameter and 1.5-μm-diameter holes. Here, MoS_2_@MoS_2_ samples exhibit smaller *E*^2D^ and larger *σ*_0_^2D^ than MoS_2_@SiO_2_ samples. The dashed lines represent the fitting curves based on Gaussian distribution. **d** Schematic illustrations of the nanoindentation process on a 2D membrane and the proposed theoretical model that considers an interfacial shear zone between the tested membrane and the substrate. The in-plane equilibrium analysis for a representative element is illustrated in the top part. *N*_*r*_ and *N*_*θ*_ are the radial and circumferential stress resultants, respectively. *r* is the distance between the selected element and the center of the hole. *dθ* is the angle of the sector. *a* is the radius of the hole. **e** Comparison between one typical experimental curve and fitted curves based on Eq. (2). The inset is the fitting curve based on *τ* = 2.795 MPa, suggesting that when the interlayer shear stress *τ* = 2.795 MPa, the fitting error reaches a minimum. **f** Distribution of fitted shear stresses that satisfies a logarithmic normal distribution. The inset shows a contour map of the nonlinear fitting error to obtain the closest shear stress of this sample.

The current fixed-boundary mechanical model for the nanoindentation test is based on the premise that the sample is firmly clamped at the edge of the hole during nanoindentation, and the measured moduli should equal the intrinsic value (180 N/m according to the literature)^27^. This premise is widely applied to SiO_2_/Si substrates, as previous studies have suggested a strong mechanical interaction between 2D materials and SiO_2_ surfaces^16–18^. However, in our experiments, this premise is challenged because the interface is MoS_2_–MoS_2_ instead of MoS_2_–SiO_2_. To test this hypothesis, we also conducted nanoindentation measurements on the suspended MoS_2_@SiO_2_ region of the same flake. Figure 3a shows the typical *F*-*δ* curves in logarithmic coordinates for MoS_2_@MoS_2_ and MoS_2_@SiO_2_ samples. The dashed lines (with the slopes of 1 and 3 in logarithmic coordinates) are plotted as indications that at small *δ*, *F* increases linearly with *δ*, which is dominated by *σ*_0_^2D^, while at larger *δ*, *F*-*δ* has a cubic relationship dominated by *E*^2D^. In this figure, the *F*-*δ* curve of the MoS_2_@SiO_2_ sample lies under that of the MoS_2_@MoS_2_ sample at first, while it surpasses the latter at larger *δ*. This result indicates that MoS_2_@SiO_2_ exhibits a larger *E*^2D^ with a smaller *σ*_0_^2D^ for this group of samples. We plotted the histograms of all measured moduli and pretension data, both well following the Gaussian distribution, as shown in Fig. 3b, c. The statistical average *E*^2D^ measured at a specific twist angle ranges from 125–153 N/m and 132–144 N/m for the MoS_2_@MoS_2_ samples over 1.0-μm-diameter holes and 1.5-μm-diameter holes, respectively, apparently lower than the measured modulus of MoS_2_@SiO_2_ (165 N/m) (Fig. 2c). This result suggests that the interface interaction and the boundary conditions of MoS_2_@MoS_2_ and MoS_2_@SiO_2_ should be different.

### Establishment of the shearing-boundary model

Considering that the Young’s modulus is an intrinsic property of a material, independent of loading and boundary conditions, the moduli of 2D materials should be constant regardless of whether the nanoindentation tests of MoS_2_ are performed on SiO_2_ or MoS_2_ substrates. The difference between the *F*-*δ* curves on different substrates is attributed to different interfacial sliding between the tested MoS_2_ layer and the SiO_2_ or MoS_2_ substrate. However, in Eq. (1), it is assumed that the tested membrane is clamped at the edge of the hole during nanoindentation, which means an infinite shear stress between the tested membrane and the substrate. It is obvious that such an assumption is not as realistic as the real experiments^40,41^. If using Eq. (1) to characterize/fit the nanoindentation curves of the same membrane on different substrates, then one might obtain different moduli when the interfacial interactions between membranes and substrates are distinct. To ensure the consistency of moduli measured from two different substrates, we developed a realistic theoretical model by considering a finite interfacial shear stress between the tested membrane and the substrate (see Fig. 3d). For simplicity, the shear stress is assumed to be constant and distributed in an annular shear zone. Such a model can be applicable to the nanoindentation of thin membranes (even ultrathin 2D materials) on any substrates. Based on this model, we derived the following analytical force–displacement relationship for an indented membrane on a given substrate:

2 $$F=	\; ({\sigma }_{0}h\pi )\delta +\,(Eh\frac{{q}^{3}}{{a}^{2}}){\delta }^{3}+\frac{1}{2}\left(1+\nu \right)\pi \tau {a}^{2}\left(\frac{\delta }{a}\right)\left[-1-\frac{{\sigma }_{0}h}{\tau a}-\frac{{C}_{\pi }{Eh}{q}^{2}}{4\tau a}{\left(\frac{\delta }{a}\right)}^{2}\right.\\ 	\left.+{\left(1+\frac{3{\sigma }_{0}h}{\tau a}+\frac{3{C}_{\pi }{Eh}{q}^{2}}{4\tau a}{\left(\frac{\delta }{a}\right)}^{2}\right)}^{\frac{1}{3}}\right]$$

where *E* is the in-plane elastic modulus in units of N/m^2^, *σ*_0_ is the pretension of the suspended membrane in units of N/m^2^, *h* is the membrane thickness, ${C}_{{\pi }}={\left(\frac{3}{{\pi }}\right)}^{\frac{2}{3}}$ is a constant and *τ* is the interfacial shear stress. In comparison to Eq. (1), the third term is the correction related to interfacial shear stress. Note that when expanding Eq. (2) via the Taylor series approximation and considering an infinite shear stress limitation (*τ* → ∞), the third term of Eq. (2) will be zero so that this equation is transformed to Eq. (1), which is consistent with our prediction. More details about the derivations of the theoretical model and Eq. (2) are given in the Supplementary Information.

We fitted the experimental force curves using the least-squares method to approach the actual value of interlayer shear stress. When we use Eq. (2) to fit the experimental curves, the modulus of MoS_2_ is fixed and taken as the measured average value from nanoindentation for MoS_2_@SiO_2_ since the modulus is a material constant. Here, we mainly extract the interlayer shear stress by using Eq. (2) to fit the experimental curve. Undoubtedly, one can extract all three parameters (including modulus, interlayer shear stress, and pretension) via the nonlinear fitting method. Figure 3e shows the comparisons between one typical experimental curve and fitted curves based on Eq. (2). When *τ* = 2.795 MPa, the fitted curve nearly coincides with the experimental curve, as evidenced by the minimum fitting error shown in the inset of Fig. 3f. We fitted all 424 nanoindentation measurement curves of the MoS_2_ monolayer with different twist angles with respect to MoS_2_@MoS_2_; the obtained values of interfacial shear stress *τ* are summarized in Fig. 3f, with a statistical average value of 2.51 MPa. We used Eq. (2) to further fit the nanoindentation measurement curves of MoS_2_@SiO_2_ and obtained an average interfacial shear stress of approximately 11.09 MPa between the MoS_2_ monolayer and the SiO_2_ substrate. This value is significantly larger than the shear stress between MoS_2_ bilayers, indicating that the previous clamped-boundary model is rational for the SiO_2_ substrate.

Moreover, we performed molecular dynamics (MD) simulations^42^ to estimate the shear stress for MoS_2_@MoS_2_ and MoS_2_@SiO_2_. In our simulations, the upper MoS_2_ monolayer is pulled along a certain direction on either a fixed MoS_2_ monolayer or amorphous SiO_2_ substrate. We took the average of the friction stress over time and obtained the average shear stress for MoS_2_@MoS_2_ and MoS_2_@SiO_2_. Details about MD simulations are supplied in the Supplementary Information. Notably, the average shear stresses (2.51 MPa and 11.09 MPa) of MoS_2_@MoS_2_ and MoS_2_@SiO_2_ from our theoretical fitting are comparable to those (4.08 MPa and 13.69 MPa) from MD simulations, respectively. We also performed density functional theory (DFT) calculations to further characterize the interlayer shear stress for MoS_2_@MoS_2_. More details are given in the Supplementary Information. The average interlayer shear stress (4.87 MPa) along the minimum energy path from our DFT calculations is close to that (4.08 MPa) from our MD simulations. These results imply that our theoretical model can be used to estimate the interfacial shear stress between a thin membrane (even for 2D materials) and a substrate. However, there exists a certain error induced by using Eq. (2) to fit the experimental results, since Eq. (2) is an approximate solution for indentation of ultrathin elastic membrane with the shearing-boundary condition. The error might mainly originate from the approximation and simplification during the derivation of Eq. (2): (i) ignoring the finite size of indenter, (ii) simplifying nonlinear distribution of interlayer shear stress between the membrane and substrate, and (iii) simplifying complex coupling/interplay among in-plane stiffness, out-of-plane deflection, pretension, and interlayer shearing.

### Twist-angle independence of interlayer shearing

**Fig. 4:**
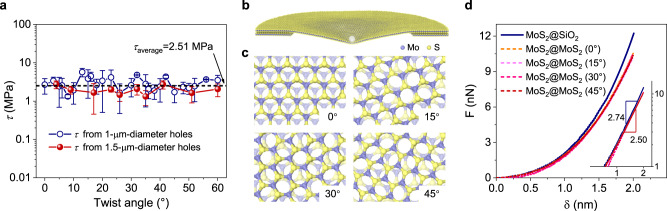
Twist-angle-independent shearing in TBLM. **a** Independence of shear stresses of TBLM samples on twist angles. Error bars represent standard deviations. The dashed line marks the average *τ* (*τ*_average_) in all TBLM samples measured over 1-μm-diameter and 1.5-μm-diameter holes. **b** Atomic configurations of the simulated system. The white sphere represents a nanoindenter with a radius of 1 nm. **c** Top views of indented MoS_2_ monolayer with different twist angles relative to the MoS_2_ substrate. **d** Nanoindentation force–displacement (*F*-*δ*) curves obtained from molecular dynamics simulations, also indicating that the interlayer mechanical interaction remains constant regardless of twist angles. The inset shows the scaling exponents of the force curves.

Having established a reliable mechanical model, we calculated the variation in shear stress by fitting the experimental data with different twist angles, as illustrated in Fig. 4a. The shear stress between the TBLM is independent of the twist angle, which is consistent with our previous hypothesis. This result can be explained by the fact that the overall interlayer vdW force is the sum of intermolecular forces, which homogenously spread over the interfaces of 2D materials. To complement the experimental results, we performed large-scale MD simulations for the nanoindentation of MoS_2_ monolayers on MoS_2_ and SiO_2_ substrates. This time, we simulated the nanoindentation process instead of the planar friction to ensure that the setup of MD simulations was very similar to the experimental configuration, as shown in Fig. 4b. We also simulated the MoS_2_ monolayer with different twist angles with respect to the MoS_2_ substrate, as illustrated in Fig. 4c. More details of the MD simulations can be found in the Supplementary Information. Figure 4d shows some typical nanoindentation curves from MD simulations. In the large displacement regime, the scaling exponents of the nanoindentation force with respect to displacement are approximately 2.50 for MoS_2_@MoS_2_ and 2.74 for MoS_2_@SiO_2_, which are close to those of the experimental curves shown in Fig. 3a and Supplementary Fig. 5b. A similar phenomenon has been captured by our theoretical model, as evidenced in Fig. 3e. Such nonlinear behavior is attributed to the common contributions of the second and third terms on the right side of Eq. (2) from our theoretical model, where the second term is the membrane stretching related to the elastic modulus and the third term is related to the interfacial shear between the membrane and the substrate. Moreover, for different twist angles, the nanoindentation curves nearly coincide with each other. This result is consistent with the experimental results shown in Supplementary Fig. 9. The reason is that both moduli related to membrane stretching (i.e., the second term in Eq. (2)) due to nanoindentation and interfacial shear (i.e., the third term in Eq. (2)) are independent of the twist angle. The above results from our theoretical model and MD simulations suggest that the interfacial shear stress between the MoS_2_ monolayer and the substrate significantly affects the nonlinear force–displacement behaviors, especially in the large deformation regime. Overall, our theoretical model considering interfacial shear stress can be used to accurately characterize the force–displacement relationship of MoS_2_ monolayers on different substrates and to ensure the consistency of moduli measured from different substrates.

To conclude, we put forward an experimental configuration and a mechanical model to quantitatively determine the interlayer mechanical interaction of 2D materials. We discovered that the measured moduli and interlayer shear stress of MoS_2_ are both independent of the twist angle. This can be attributed to the long-range interaction of vdW forces that homogenously spread over the interfaces of 2D materials. The shear stresses of the MoS_2_–MoS_2_ interface and MoS_2_–SiO_2_ interface are 2.51 and 11.09 MPa according to our experiments, which coincide well with the values obtained from MD simulations (4.08 and 13.69 MPa, respectively). Our strategy can be facilely applied to probe the interlayer mechanical interactions of other 2D systems and sheds light on experimentally obtaining the interfacial shear stress between 2D material interfaces.

## Methods

### Synthesis of MoS_2_ monolayers

MoS_2_ monolayers were grown on SiO_2_/Si substrates under ambient pressure in a chemical vapor deposition (CVD) system. A piece of SiO_2_/Si substrate treated in piranha solution (H_2_SO_4_:H_2_O_2_ = 3:1) was faced down and placed on a quartz boat filled with MoO_3_ powder. A droplet of PTAS solution was used as a seeding promoter. PTAS was synthesized by the alkaline hydrolysis of perylene-3,4,9,10-tetracarboxylic dianhydride (PTCDA). At first, KOH aqueous solution was added into the mixture of PTCDA and ethanol. Then, the reaction mixture was refluxed for 20 h. At last, the final product PTAS was filtrated out after ethyl ether was added to the solution^43^. Before heating, the whole CVD system was purged with 200 sccm Ar for 10 min. Then, the temperature of MoO_3_ was ramped to 650 °C at a rate of 15 °C/min  and maintained at 650 °C for 3 min with 5 sccm Ar. The temperature of sulfur powder heated by a heating belt was ramped to 180 °C at a rate of 30 °C/min as soon as the temperature of MoO_3_ reached 500 °C. After growth, the furnace was opened for rapid cooling.

### Preparation of TBLM

For the wet transfer, a layer of PMMA was spin-coated with a speed of 2500 rpm on a SiO_2_/Si substrate with triangular MoS_2_ monolayers, which were used as bottom MoS_2_ monolayers (Fig. 1a). Then, the PMMA/MoS_2_ layer was etched away from the substrate in 1 M KOH solution. After rinsed with ultrapure water three times, the PMMA/MoS_2_ layer was picked up by a clean holey substrate that was pre-etched by UV photolithography and dry-etched into patterns of holes with a diameter of ~1 μm or ~1.5 μm and a periodic interval of 2.5 μm. The holey substrate with the PMMA/MoS_2_ layer was then heated at 180 °C for 1 min and immersed in acetone at 80 °C for 2 h to remove PMMA. After that, the MoS_2_ monolayers over the holes collapsed. Finally, the holey substrate with the MoS_2_ monolayers was annealed at 350 °C in vacuum (1 × 10^−3 ^Pa) to further remove any PMMA residues.

For the dry transfer, an atomically flat Si wafer was used as a supporting substrate to cure PDMS, which avoids the large surface roughness of PDMS that may induce wrinkles on MoS_2_ monolayers. The cured PDMS was then cut into a small piece and adhered onto a glass slide, and the glass slide/PDMS was further attached to a SiO_2_/Si substrate with triangular MoS_2_ monolayers, which were used as upper MoS_2_ monolayers (Fig. 1a). The glass slide/PDMS/MoS_2_ was removed from the SiO_2_/Si substrate by immersing them in ultrapure water for one hour. Then, the glass slide/PDMS/MoS_2_ was aligned with and adhered onto the bottom MoS_2_ monolayers on the holey substrate by homemade transfer equipment in an Ar glove box. After heating at 60 °C for 15 min, the glass slide/PDMS was lifted upwards, leaving the upper MoS_2_ monolayers stacked on top of the bottom MoS_2_ monolayers and forming TBLM with random twist angles. Finally, the TBLM sample was annealed at 350 °C in vacuum (1 × 10^−3 ^Pa) to remove PDMS residues on the surface and ensure that the twisted bilayer interacted effectively.

### Characterizations

An optical microscope (OLYMPUS BX51 M) was used to find MoS_2_ flakes and measure the twist angles of TBLM. Atomic force microscopy (AFM, Bruker Multimode 8) was used to measure surface topology and conduct nanoindentation tests. Raman spectra were obtained by a spectrometer (Horiba iHR550) using an excitation laser with a wavelength of 532 nm. ADF-STEM images of Moiré patterns were obtained on a JEM-ARM200CF operated at an acceleration voltage of 200 kV, with a collection angle of 40–160 mrad.

### Nanoindentation

Before indentation, the sample was scanned in tapping mode under AFM until the thermal drift was negligible. Then, the AFM tip was positioned at the center of a suspended membrane. With the sample stage moving upwards by a distance of *z*, a point force *F*_0_ was applied to the sample. Upon reaching the preinstalled force, the sample stage moved downwards to release the force. After each series of nanoindentation tests, we scanned the sample again to detect any possible slippery.

### MD simulations

MD simulations were performed via the large-scale atomic/molecular massively parallel simulator (LAMMPS)^42^. The details of the MD simulations are provided in the Supplementary Information.

### DFT calculations

DFT calculations were performed via VASP^44^ to characterize the interlayer shear stress for bilayer MoS_2_. The details of the DFT calculations are provided in the Supplementary Information.

## Supplementary information

Supplementary Information

Peer Review File

## Data Availability

Relevant data supporting the key findings of this study are available within the article and the Supplementary Information file. All raw data generated during the current study are available from the corresponding authors upon request.

## References

[1] Geim AK, Grigorieva IV (2013). Van der Waals heterostructures. Nature.

[2] Liu Y (2016). Van der Waals heterostructures and devices. Nat. Rev. Mater..

[3] Tan C (2017). Recent advances in ultrathin two-dimensional nanomaterials. Chem. Rev..

[4] Liu K (2014). Evolution of interlayer coupling in twisted molybdenum disulfide bilayers. Nat. Commun..

[5] van der Zande AM (2014). Tailoring the electronic structure in bilayer molybdenum disulfide via interlayer twist. Nano Lett..

[6] Liao M (2018). Twist angle-dependent conductivities across MoS_2_/graphene heterojunctions. Nat. Commun..

[7] Yan W (2019). Probing angle-dependent interlayer coupling in twisted bilayer WS_2_. J. Phys. Chem. C..

[8] Cao Y (2018). Unconventional superconductivity in magic-angle graphene superlattices. Nature.

[9] Tran K (2019). Evidence for moire excitons in van der Waals heterostructures. Nature.

[10] Cao Y (2020). Tunable correlated states and spin-polarized phases in twisted bilayer-bilayer graphene. Nature.

[11] Hu G (2020). Topological polaritons and photonic magic angles in twisted alpha-MoO_3_ bilayers. Nature.

[12] Jin C (2019). Observation of moire excitons in WSe_2_/WS_2_ heterostructure superlattices. Nature.

[13] Chen W (2019). Direct observation of van der Waals stacking-dependent interlayer magnetism. Science.

[14] Wang B (2020). Bioelectronics related 2D materials beyond graphene: fundamentals, properties, and applications. Adv. Funct. Mater..

[15] Liu K, Wu JQ (2016). Mechanical properties of two-dimensional materials and heterostructures. J. Mater. Res..

[16] Lloyd D (2017). Adhesion, stiffness, and instability in atomically thin MoS_2_ bubbles. Nano Lett..

[17] Wang G (2017). Measuring interlayer shear stress in bilayer Graphene. Phys. Rev. Lett..

[18] Koenig SP, Boddeti NG, Dunn ML, Bunch JS (2011). Ultrastrong adhesion of graphene membranes. Nat. Nanotechnol..

[19] Manzanares-Negro Y (2020). Improved graphene blisters by ultrahigh pressure sealing. ACS Appl. Mater. Interfaces.

[20] Li B, Liu X, Guo W (2020). Probing interactions at two-dimensional heterointerfaces by boron nitride-wrapped tip. Nano Res..

[21] Li B (2019). Probing van der Waals interactions at two-dimensional heterointerfaces. Nat. Nanotechnol..

[22] Liu Y (2018). Interlayer friction and superlubricity in single-crystalline contact enabled by two-dimensional flake-wrapped atomic force microscope tips. ACS Nano.

[23] Liu K (2014). Elastic properties of chemical-vapor-deposited monolayer MoS_2_, WS_2_, and their bilayer heterostructures. Nano Lett..

[24] López-Polín G (2014). Increasing the elastic modulus of graphene by controlled defect creation. Nat. Phys..

[25] Falin A (2017). Mechanical properties of atomically thin boron nitride and the role of interlayer interactions. Nat. Commun..

[26] Castellanos-Gomez A (2012). Elastic properties of freely suspended MoS_2_ nanosheets. Adv. Mater..

[27] Simone Bertolazzi JB, Kis A (2011). Stretching and breaking of ultrathin MoS_2_. ACS Nano.

[28] Lipatov A (2018). Elastic properties of 2D Ti_3_C_2_T_x_ MXene monolayers and bilayers. Sci. Adv..

[29] Lv R (2015). Transition metal dichalcogenides and beyond: synthesis, properties, and applications of single- and few-layer nanosheets. Acc. Chem. Res..

[30] Wang B (2020). Bifunctional NbS_2_-based asymmetric heterostructure for lateral and vertical electronic devices. ACS Nano.

[31] Lee YH (2012). Synthesis of large-area MoS_2_ atomic layers with chemical vapor deposition. Adv. Mater..

[32] Sun Y (2019). Elastic properties and fracture behaviors of biaxially deformed, polymorphic MoTe_2_. Nano Lett..

[33] Li X-L (2017). Layer-number dependent optical properties of 2D materials and their application for thickness determination. Adv. Funct. Mater..

[34] Lee C (2010). Anomalous lattice vibrations of single- and few-layer MoS_2_. ACS Nano.

[35] Wan KT, Guo S, Dillard DA (2003). A theoretical and numerical study of a thin clamped circular film under an external load in the presence of a tensile residual stress. Thin Solid Films.

[36] Komaragiri U, Begley MR (2005). The mechanical response of freestanding circular elastic films under point and pressure loads. J. Appl. Mech.-T Asme..

[37] Lee C, Wei X, Kysar JW, Hone J (2008). Measurement of the elastic properties and intrinsic strength of monolayer graphene. Science.

[38] Vella D, Davidovitch B (2017). Indentation metrology of clamped, ultra-thin elastic sheets. Soft Mater..

[39] Ares, P. et al. Van der Waals interaction affects wrinkle formation in two-dimensional materials. *Proc. Natl. Acad. Sci*. **118**, e2025870118 (2021). 10.1073/pnas.2025870118 PMC8040643 33790019

[40] Davidovitch B, Guinea F (2021). Indentation of solid membranes on rigid substrates with van der Waals attraction. Phys. Rev. E.

[41] Dai Z, Lu N (2021). Poking and bulging of suspended thin sheets: Slippage, instabilities, and metrology. J. Mech. Phys. Solids.

[42] Plimpton S (1995). Fast parallel algorithms for short-range molecular-dynamics. J. Comput. Phys..

[43] Zhang XQ, Lin CH, Tseng YW, Huang KH, Lee YH (2015). Synthesis of lateral heterostructures of semiconducting atomic layers. Nano Lett..

[44] Kresse G, Furthmüller J (1996). Efficient iterative schemes for ab initio total-energy calculations using a plane-wave basis set. Phys. Rev. B: Condens. Matter.

