# Supplementary material for: Determining the interlayer shearing in twisted bilayer MoS2 by nanoindentation
Source: Nat Commun. 2022 Jul 6;13:3898. doi: 10.1038/s41467-022-31685-7 (PMC9259563; doi:10.1038/s41467-022-31685-7)
Supplement: Supplementary file 1 — Supplementary Information [file 41467_2022_31685_MOESM1_ESM.pdf]

Supplementary Information for

Determining the Interlayer Shearing in Twisted Bilayer MoS_2 by Nanoindentation

Yufei Sun[1,#], Yujia Wang[2,#], Enze Wang[1,#], Bolun Wang[1,#], Hengyi Zhao[2], Yongpan Zeng[2], Qinghua Zhang[3], Yonghuang Wu[1], Lin Gu[3], Xiaoyan Li[2,*], Kai Liu[1,*]

[1] State Key Laboratory of New Ceramics and Fine Processing & Key Laboratory of Advanced Materials of Ministry of Education, School of Materials Science and Engineering, Tsinghua University, Beijing 100084, China

[2] Center for Advanced Mechanics and Materials, Applied Mechanics Laboratory, Department of Engineering Mechanics, Tsinghua University, Beijing 100084, China

[3] Institute of Physics, Chinese Academy of Sciences, Beijing 100190, China

* Corresponding authors. E-mail: liuk@tsinghua.edu.cn (K.L.);
xiaoyanlithu@tsinghua.edu.cn (X.L.)

# These authors contributed equally to this work.

Keywords: MoS_2, twist angle, nanoindentation, interlayer shearing

1. High quality and clean interfaces of TBLM samples

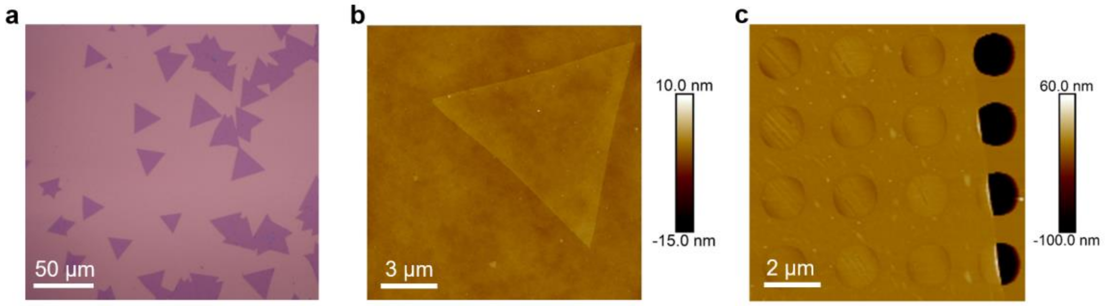

Supplementary Fig. 1 As-grown (a) and transferred (b, c) MoS_2 monolayers. Clean surfaces and few bubbles/wrinkles can be observed on MoS_2 monolayers after either optimized PMMA-assistant wet transfer (b) or optimized PDMS-assistant dry transfer (c).

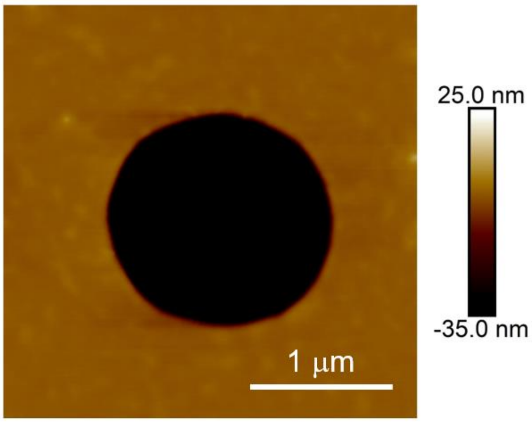

Supplementary Fig. 2 AFM image of a broken bottom MoS_2 monolayer over a hole on a SiO_2/Si substrate.

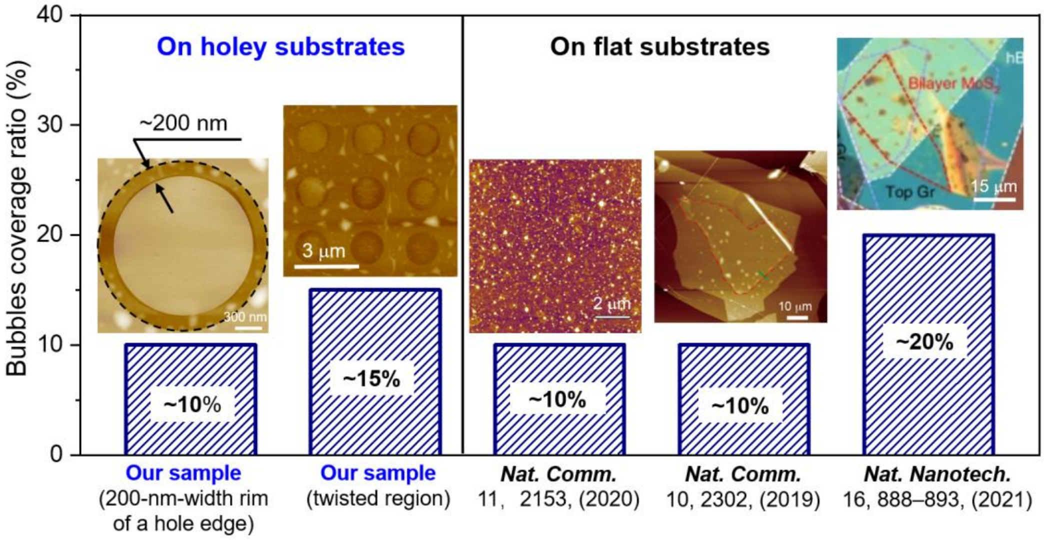

Supplementary Fig. 3 Comparison of our TBLM samples on holey substrates with other twisted 2D materials on flat substrates reported in literature. A very low density of bubbles and wrinkles is observed in the TBLM samples on holey substrates. Note that in our nanoindentation experiment, the suspended upper MoS_2 monolayer is clamped somewhere around a hole edge according to the fixed-boundary model or shearing-boundary model. Therefore, the measured moduli would strongly depend on the tightness of the twisted region around the hole edge. This region relatively lacks bubbles and wrinkles because they may merge and move inside the hole. As shown in the most left panel of this figure, the bubbles coverage ratio of the TBLM sample in the 200-nm-width rim of a hole edge is only ~10%, reaching the lows reported on flat substrates.

2. Experimental characterization of interlayer mechanical interactions

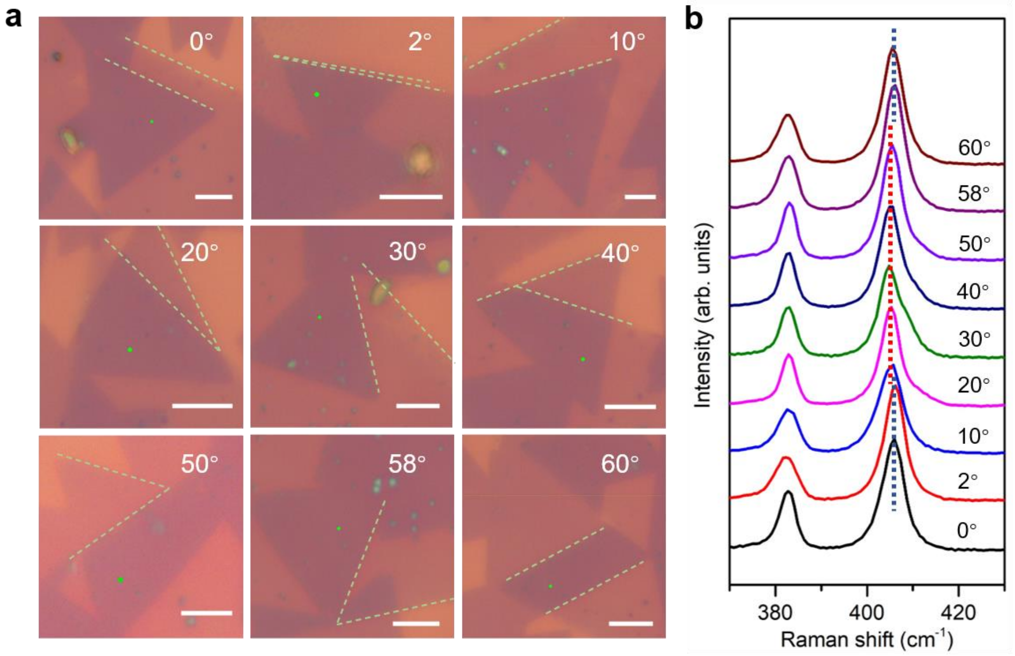

Supplementary Fig. 4 Raman characterization of interlayer interaction. (**a**) Optical image of TBLM transferred on normal SiO_2/Si substrate, where the twist angles can be directly measured. Scale bars, 5 μm. (**b**) The corresponding Raman spectra of the samples. When the twist angle is close to 0 or 60°, the peak interval reaches maximum; at other twist angles, the peak intervals are smaller and remain constant.

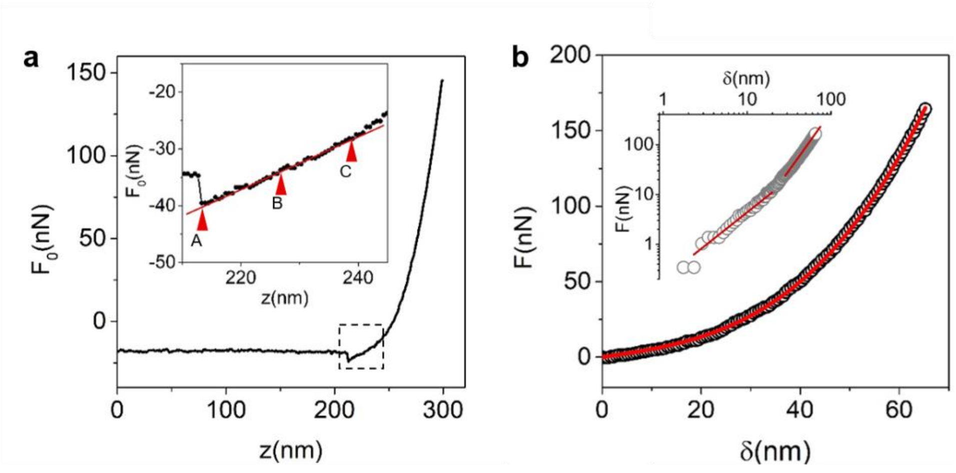

Supplementary Fig. 5 Typical F_0-z curve and the corresponding F-δ curve. (**a**) A typical F_0-z curve with the linear part shown in the inset (where F_0 is the absolute load). At point A, the tip contacts the membrane; at point C the tip begins to deform the membrane. Point B is the midpoint of AC, which is set as the zero point of F-δ curve.

(**b**) The corresponding *F*-δ curve. Inset shows the *F*-δ curve in logarithmic coordinate, with the red lines showing its gradient. At small δ, the gradient is close to 1 while at larger δ the gradient approaches 3, which fits well with Eq. (1).

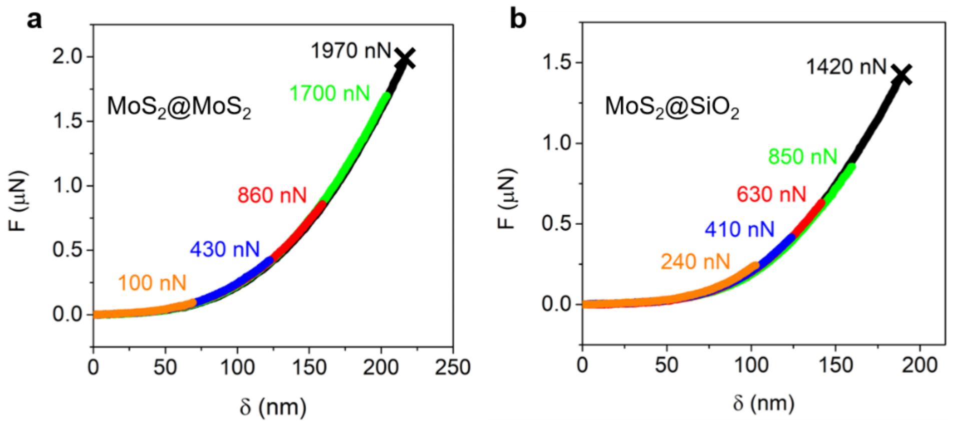

Supplementary Fig. 6 *F*-δ curves obtained in five consecutive nanoindentations under different loads. The *F*-δ curves of MoS_2@MoS_2 (a) or MoS_2@SiO_2 (b) follow the nearly identical trace and still obey the cubic relationship (F~δ^3) near the breaking force, indicating a brittle fracture behavior and also very good reproductivity of our measurements.

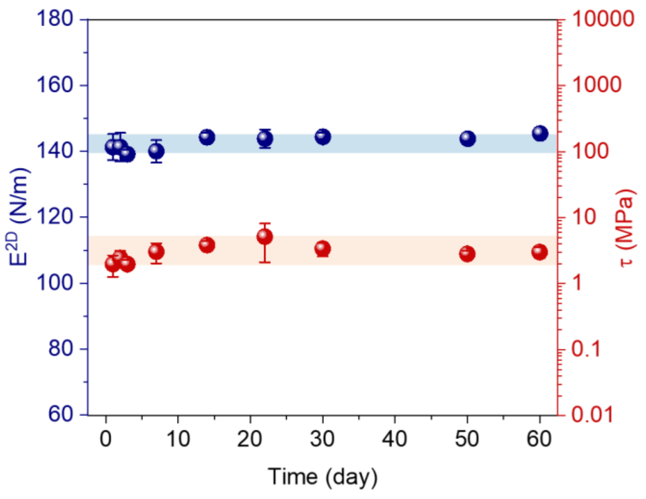

Supplementary Fig. 7 Variations of E^{2D} and τ with time in two months.

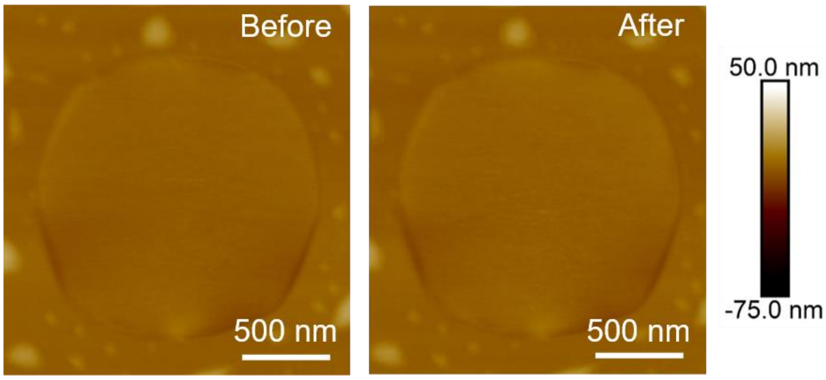

Supplementary Fig. 8 AFM images of a suspended upper MoS_2 monolayer before and after nanoindentation. The identical surface topology suggests no plastic deformation of the MoS_2 monolayer during the nanoindentation.

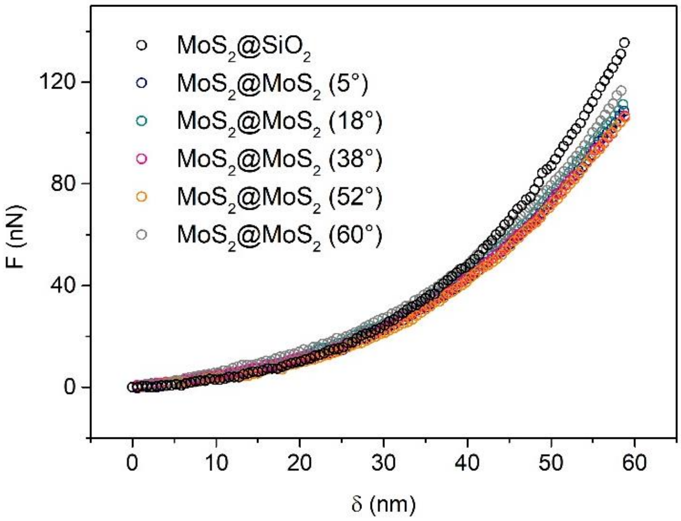

Supplementary Fig. 9 Typical *F*-*δ* curves of MoS_2@SiO_2 and MoS_2@MoS_2 at various twist angles. The force curves of MoS_2@MoS_2 coincide with each other regardless of the twist angle, while the force curve of MoS_2@SiO_2 surpasses these curves at larger δ, showing a larger measured elastic modulus. Since the force curve is influenced by the radius of the hole, the curves shown here are chosen from the samples over the holes with the same radii (a=0.5 μm).

3. Theoretical model of nanoindentation of 2D materials on the substrate

Previous theoretical studies on nanoindentation test of 2D materials assumed clamped boundary conditions at the edge of the hole[1-3]. Such assumption is not as realistic as the real experimental conditions. Recently, the interfacial shear has been taken into account in pressurized graphene blisters to characterize the interactions between graphene and substrate[4]. Considering an interfacial shear between indented membrane and substrate in the supported region, we present an analytical model of a circular membrane under point load. This model can be used to describe the force-displacement relationship of nanoindentation of membrane (even 2D materials). As shown in Fig. 3d, the membrane in our model is divided into two parts: one is the membrane (under plane-stress state) subjected to interfacial shear stress outside the hole ($r>a$, a is the radius of the hole), and another is the membrane subjected to applied point load in suspended region ($r<a$). It is noted that two parts should satisfy the continuity conditions of displacement and stress in the radial direction at the edge of the hole ($r=a$).

In the supported region ($r>a$), we assume for simplicity that the interfacial shear stress τ is constant and distributed in an annular interfacial shear zone ($a<r<\rho a$), as shown in Fig. 3d. The in-plane stress decays to zero at the position $r=\rho a$, where ρ is dimensionless and can be determined by a specific applied load and corresponding displacement. It is noted that ρ is obviously greater than 1 and approaches 1 at the limit of $\tau \to \infty$ (for clamped boundary conditions). The in-plane equilibrium analysis in Fig. 3d gives,

$$\left(N_r + \mathrm{d}N_r\right)\left(r + \mathrm{d}r\right)\mathrm{d}\theta + \tau r \mathrm{d}\theta \mathrm{d}r - N_r r \mathrm{d}\theta - 2N_\theta \mathrm{d}r \frac{\mathrm{d}\theta}{2} = 0 \tag{1}$$

Neglecting the higher order terms, we obtained,

$$N_r + r\frac{\mathrm{d}N_r}{\mathrm{d}r} - N_\theta + \tau r = 0 \tag{2}$$

where N_r and N_θ are the radial and circumferential stress resultants, respectively. For the MoS_2 membrane with the thickness of h, $N_r = \sigma_r h$ and $N_\theta = \sigma_\theta h$, where σ_r and σ_θ are the radial and circumferential stresses, respectively. The linear elastic stress-strain relationship gives,

$$E\varepsilon_r = \sigma_r - \nu\sigma_\theta \tag{3}$$

$$E\varepsilon_\theta = \sigma_\theta - \nu\sigma_r \tag{4}$$

where E is the Young's modulus and ν is the Poisson's ratio. The relations of the strain and displacement are given by,

$$\varepsilon_r = \frac{\mathrm{d}u}{\mathrm{d}r} + \frac{1}{2}\left(\frac{\mathrm{d}w}{\mathrm{d}r}\right)^2 \tag{5}$$

$$\varepsilon_\theta = \frac{u}{r} \tag{6}$$

where u and w are the radial and transversal displacements, respectively. Note that w=0 in the supported region, thus the second term on the right side of Eq. (5) vanishes. Substituting Eqs. (3)-(6) into Eq. (2) gives,

$$r\frac{\mathrm{d}^2N_r}{\mathrm{d}r^2}+3\frac{\mathrm{d}N_r}{\mathrm{d}r}+(2+\nu)\tau=0 \tag{7}$$

The boundary conditions at *r*=*ρa* are expressed as $N_r=N_\theta=0$. Thus, we can obtain the radial and circumferential resultant stresses as,

$$N_r=-\frac{2+\nu}{3}\tau r+\frac{1-\nu}{6}\rho^3\tau a^3\frac{1}{r^2}+\frac{1+\nu}{2}\rho\tau a \tag{8}$$

$$N_\theta=-\frac{1+2\nu}{3}\tau r-\frac{1-\nu}{6}\rho^3\tau a^3\frac{1}{r^2}+\frac{1+\nu}{2}\rho\tau a \tag{9}$$

Substituting the above solutions to Eqs. (4) and (6), the radial displacement at *r*=*a* is obtained as,

$$u^{out}|_{r=a}=\frac{(1-\nu^2)a^2\tau}{Eh}\left(-\frac{1}{6}\rho^3+\frac{1}{2}\rho-\frac{1}{3}\right) \tag{10}$$

where ρ can be determined by continuity conditions. Note that $u^{out}|_{r=a}$ is negative when ρ is greater than 1, meaning that the direction of in-plane displacement at the edge of hole is inward.

In the suspended region (*r*<*a*), previous studies proposed an expression for the force-displacement relationship of a clamped elastic thin membrane under point load,

$$F=\pi\sigma_0 ha\left(\frac{\delta}{a}\right)+q^3Eha\left(\frac{\delta}{a}\right)^3 \tag{11}$$

where *F* is the applied force, σ_0 is the pretension stress, δ is the deflection at the

center point and q is parameter related to the Poisson's ratio ν and expressed as $q=1/\left(1.049-0.15\nu-0.16\nu^2\right)$. Eq. (11) contains two terms separately from two special cases: the first term is associated with the pretension when the load is small, while the second term reflects the large displacement, i.e., $\delta \gg h$. For the case of second term, the radial stress is proportional to $\left(r/a\right)^{-\frac{2}{3}}$ and expressed as[2],

$$N_r=\frac{C_\pi Ehq^2}{4}\left(\frac{\delta}{a}\right)^2\left(\frac{r}{a}\right)^{-\frac{2}{3}} \tag{12}$$

where $C_\pi=\left(\frac{3}{\pi}\right)^{\frac{2}{3}}$ is a constant. For Eq. (11), the fix boundary conditions at the edge are assumed. Such assumption is not realistic and obviously cannot satisfy the continuity conditions of radial displacement at the edge of the hole. Therefore, we introduce the third term to satisfy the continuity conditions in terms of radial stress and displacement. Considering a homogeneous stress field independent of r, i.e., $\sigma_r=\sigma_\theta=\sigma_c<0$, the corresponding strain is given by,

$$\varepsilon_\theta=\frac{1}{E}\left(\sigma_\theta-\nu\sigma_r\right)=\frac{\left(1-\nu\right)\sigma_c}{E} \tag{13}$$

According to the strain-displacement relationship $\varepsilon_\theta=u/r$, the radial displacement field is obtained as,

$$u^{in}=\varepsilon_\theta r=\frac{\left(1-\nu\right)\sigma_c}{E}r \tag{14}$$

The radial displacement at $r=a$ is determined by,

$$u^{in}|_{r=a} = \frac{(1-\nu)\sigma_c a}{E} \tag{15}$$

Subsequently, we will use the continuity conditions of radial displacement and stress at the edge of the hole to determine the stress field σ_c.

Both radial displacement and stress are continuous at the edge of hole, i.e., $u^{in}|_{r=a} = u^{out}|_{r=a}$ and $N_r^{in}|_{r=a} = N_r^{out}|_{r=a}$. Combining Eqs. (8) and (10) outside the hole, and Eqs. (12) and (15) for suspended membrane over the hole, we obtained the following continuity conditions,

$$\frac{(1-\nu)\sigma_c a}{E} = \frac{(1-\nu^2)a^2\tau}{Eh}\left(-\frac{1}{6}\rho^3 + \frac{1}{2}\rho - \frac{1}{3}\right) \tag{16}$$

$$\sigma_0 h + \frac{C_\pi Ehq^2}{4}\left(\frac{\delta}{a}\right)^2 + \sigma_c h = \left(-\frac{2+\nu}{3} + \frac{1-\nu}{6}\rho^3 + \frac{1+\nu}{2}\rho\right)\tau a \tag{17}$$

Solving Eqs. (16) and (17), we obtained the expressions of ρ and stress σ_c,

$$\rho = \left(1 + \frac{3\sigma_0 h}{\tau a} + \frac{3C_\pi Ehq^2}{4\tau a}\left(\frac{\delta}{a}\right)^2\right)^{\frac{1}{3}} \tag{18}$$

$$\sigma_c = \frac{(1+\nu)\tau a}{2h}\left[-1 - \frac{\sigma_0 h}{\tau a} - \frac{C_\pi Ehq^2}{4\tau a}\left(\frac{\delta}{a}\right)^2 + \left(1 + \frac{3\sigma_0 h}{\tau a} + \frac{3C_\pi Ehq^2}{4\tau a}\left(\frac{\delta}{a}\right)^2\right)^{\frac{1}{3}}\right] \tag{19}$$

Note that the homogeneous stress σ_c resembles the pretension stress, thus the relationship of force and deflection due to σ_c can be expressed as $F^{\sigma_c} = \pi\sigma_c ha\left(\frac{\delta}{a}\right)$.

Therefore, summing two terms in Eq. (11) and the term related to σ_c, we finally obtained the force-displacement relationship by considering interfacial sliding in the supported region,

$$F=\pi\sigma_0 ha\left(\frac{\delta}{a}\right)+q^3Eha\left(\frac{\delta}{a}\right)^3 +\frac{1}{2}(1+\nu)\pi\tau a^2\left(\frac{\delta}{a}\right)\left[-1-\frac{\sigma_0 h}{\tau a}-\frac{C_\pi Ehq^2}{4\tau a}\left(\frac{\delta}{a}\right)^2+\left(1+\frac{3\sigma_0 h}{\tau a}+\frac{3C_\pi Ehq^2}{4\tau a}\left(\frac{\delta}{a}\right)^2\right)^{\frac{1}{3}}\right] \tag{20}$$

By expanding the fourth term in the brackets on the right side of Eq. (19) via the Taylor series approximation with two-order accuracy, we obtained the approximate expression of stress σ_c as,

$$\sigma_c \approx -\frac{(1+\nu)a}{18\tau h}\left(\frac{3\sigma_0 h}{a}+\frac{3C_\pi Ehq^2}{4a}\left(\frac{\delta}{a}\right)^2\right)^2 \tag{21}$$

It is noted from Eq. (21) that the value of σ_c tends to zero as $\tau\to\infty$, meaning that Eq. (20) can be reduced to Eq. (11) at the limit of $\tau\to\infty$. We used Eq. (20) to estimate the shear stress between MoS_2 monolayer and MoS_2 and SiO_2 substrates by fitting the experimental nanoindentation force-displacement curves. The obtained average values of interfacial shear stress between MoS_2 and MoS_2 or SiO_2 substrate is 2.51 MPa and 11.09 MPa, respectively.

4. Atomistic simulations for nanoindentation of MoS_2 monolayers on substrates

To complement the experimental results, we performed a series of large-scale molecular dynamics (MD) simulations for nanoindentation of MoS_2 monolayer

spanning over the open hole of MoS_2 and SiO_2 substrates. All MD simulations were performed via the large-scale atomic/molecular massively parallel simulator (LAMMPS)[5]. Similar to the experimental nanoindentation set-up, we constructed the simulated systems, including a rigid nanoindenter with the radius of 1 nm and a circular MoS_2 monolayer with the radius of 15 nm spanning over the hole with the radius of 10 nm, as illustrated in Fig. 4a. The outermost circular ribbon with the width of 1 nm is always fixed. The hole is located in the MoS_2 or amorphous SiO_2 substrate. The SiO_2 substrate is generated by a melting-quenching process[6]. During this process, the temperature of system first increases gradually from 300 K to 5000 K within 100 ps, then is kept at 5000 K for full relaxation for 1 ns and finally decreases to 300 K with a rapid cooling rate of about 6 K/ps. The system was first equilibrated by the energy minimization and followed relaxation for 100 ps at 300 K via an NVT ensemble. After equilibration, the rigid indenter was placed on the center of MoS_2 monolayer, and then moved downward with a constant velocity of 1 m/s. During simulations, the reactive many-body potential[7] and the Tersoff potential[8] are used to describe the interatomic interactions in MoS_2 and SiO_2 system, respectively. The interaction between indented membrane and substrate is described by the 6-12 Lennard-Jones potentials[9,10] with a series of parameters listed in Supplementary Table 1. The indentation force on each atom of indented membrane is calculated by,

$$F_{\text{ind}}(r) = \begin{cases} -K(r-R)^2 & r \le R \\ 0 & r > R \end{cases} \tag{21}$$

where r is the distance between the center of indenter and the atom, R is the indenter

radius, and K represents a spring stiffness and is set as 10 eV/Å^3. To reduce the influence of thermal fluctuation, the indentation force between indenter and membrane is obtained by summing the indentation forces on all the atoms and then taking average over each time for indenter's moving downward for 1 Å. To investigate the influence of twist angle of indented MoS_2 on the nanoindentation, we rotated the indented monolayer by 15º, 30º, and 45º with respect to the substrate during construction of MoS_2 monolayer on MoS_2 substrate, as illustrated in Fig. 4c.

5. Atomistic simulations for MoS_2 monolayers sliding on substrates

To estimate the shear stress between MoS_2 membrane and substrate, we performed MD simulations for the sliding of MoS_2 monolayer on MoS_2 or SiO_2 substrate via LAMMPS[5]. We first constructed two simulated systems: one is the MoS_2 monolayer (with in-plane size of 20.33×20.28 nm^2) covering on the MoS_2 monolayer with the same size, and another is the MoS_2 monolayer (with in-plane size of 29.54×33.46 nm^2) covering on the SiO_2 block with a dimension of 29.54×33.46×5 nm^3 (see insets in Supplementary Fig. 10a and 10b). Both MoS_2 and SiO_2 substrates are fixed during simulations. The simulated systems were first equilibrated by the energy minimization and followed dynamic relaxation at 300 K for 100 ps. After equilibration, we used a spring with the stiffness of 801.0 N/m to pull the MoS_2 monolayer on the substrates along the given direction via steered MD, as shown in the insets in Supplementary Fig. 10a and 10b. During simulations, we calculated the force acting on MoS_2 monolayer from the substrate along the opposite direction of its motion as the friction force. Supplementary Fig. 10a and 10b show the variation of friction force of MoS_2 monolayer

on the MoS_2 and SiO_2 substrate with the time, respectively. It is seen in Supplementary Fig. 10a that there exists the significant force fluctuation with a certain periodicity. Such force fluctuation is attributed to the lattice commensurance between MoS_2 monolayer and MoS_2 substrate[11]. However, the force fluctuation in Supplementary Fig. 10b is not pronounced and has no obvious periodicity, because the SiO_2 substrate is amorphous so that there is no strong commensurance between MoS_2 monolayer and SiO_2 substrate. We took average of the friction force over time, then divided by the effective area, and obtained the average shear stress for MoS_2-MoS_2 and MoS_2-SiO_2 as 4.08 MPa and 13.69 MPa, respectively.

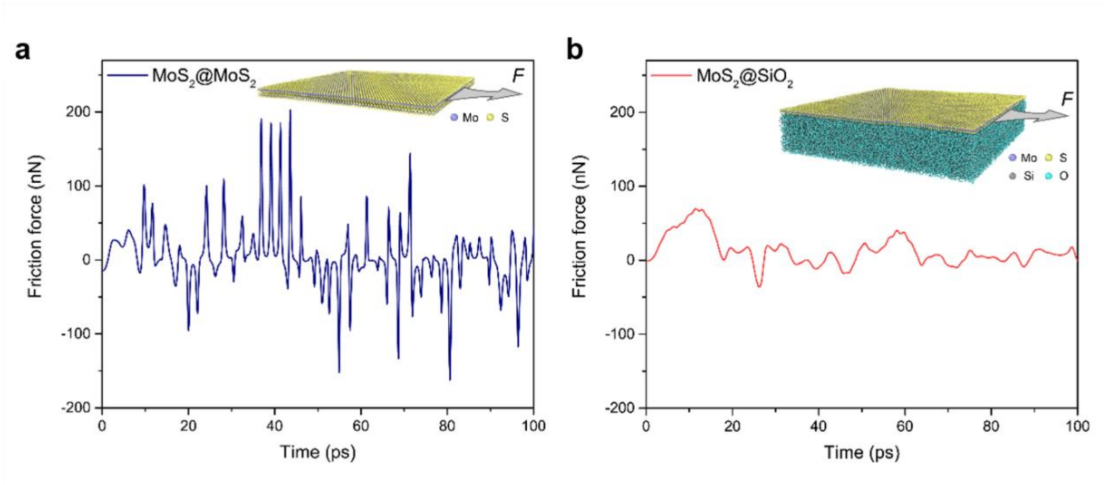

Supplementary Fig. 10 Atomistic simulations of MoS_2 monolayer sliding on MoS_2 and SiO_2 substrates. (**a**) Variation of friction force of MoS_2 monolayer sliding on MoS_2 substrate with the time. The significant fluctuation of friction force is due to the lattice commeasure between MoS_2 monolayer and MoS_2 substrate. (**b**) Variation of friction force of MoS_2 monolayer sliding on MoS_2 substrate with sliding distance. The insets in (a) and (b) show the simulated atomic configurations of MoS_2 monolayer being stretched for sliding on MoS_2 and SiO_2 substrates.

6. DFT calculations for interlayer shear for bilayer MoS_2

We performed the density function theory (DFT) calculations via VASP[12] to characterize the interlayer shear for bilayer MoS_2. The generalized gradient approximation (GGA) of Perdew- Burke- Ernzerhof (PBE) functional[13] is adopted to describe the exchange-correlation energy. The van der Waals interaction between two MoS_2 layers is considered by means of the Grimme correction[14]. The simulated system is a hexagonal cell of bilayer MoS_2, which includes one MoS_2 unit per layer (Supplementary Fig. 11). The dimension along the height direction of cell is enlarged to introduce at least 30 Å of vacuum layer. A 12×12×1 *k*-points grid is used for the sampling of the Brillouin zone. The kinetic energy cutoff is set as 550 eV. To mimic the sliding, we rigidly moved the upper layer to the specific positions along the armchair direction or minimum energy path (MEP)[15]. These positions are marked by red dots in Supplementary Fig. 11. We calculated the energy change ΔE of simulated system after sliding with respect to initial equilibrium position. Then, the work of separation ΔW_{sep} is obtained by dividing the energy change ΔE by the unit cell area[15], i.e., $\Delta W_{sep} = \Delta E/A$. The work of separation reflects the energy per unit area required to separate two layers in bilayer MoS_2. Supplementary Fig. 12a and b show the variation of work of separation with the sliding distance along the armchair direction and MEP, respectively. The results from our DFT calculations agree well with those from previous DFT calculations[15] via QUANTUM ESPRESSO. The friction force f is obtained by differentiating the energy change with respect to the sliding distance[15], i.e., $f = -\frac{d\Delta E}{dr}$. Supplementary Fig. 12c and d show the variation of friction force with the sliding distance along the armchair

direction and MEP, respectively. We took average of the friction force over the sliding distance, then divided by the unit cell area, and obtained the average interlayer shear stress for bilayer MoS_2 for armchair direction and MEP as 8.81 MPa and 4.87 MPa, respectively. These values of average interlayer shear stress are close to that (4.08 MPa) from our MD simulations.

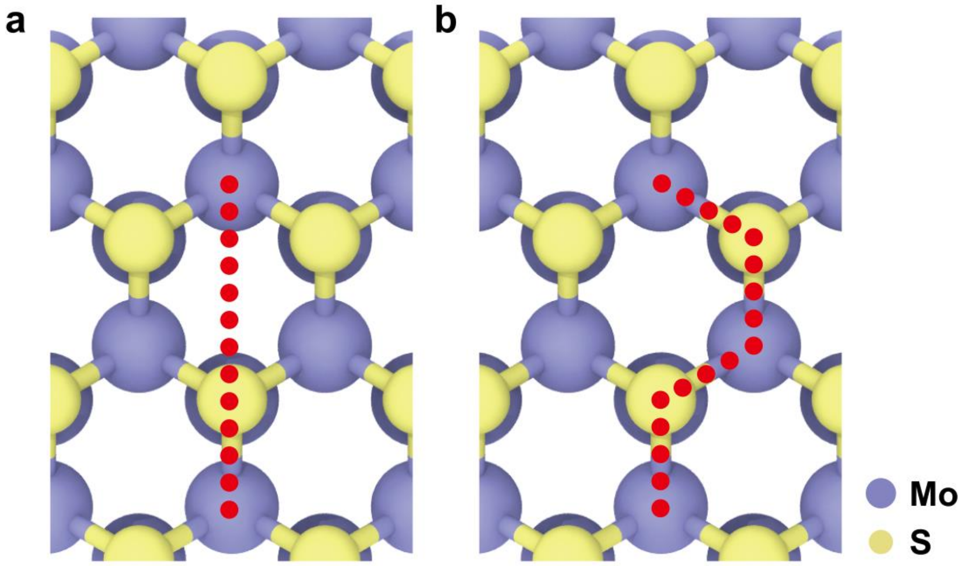

Supplementary Fig. 11 Top view of simulated system and sliding path. (**a**) Sliding of upper layer along the armchair direction. (**b**) Sliding of upper layer along the MEP.

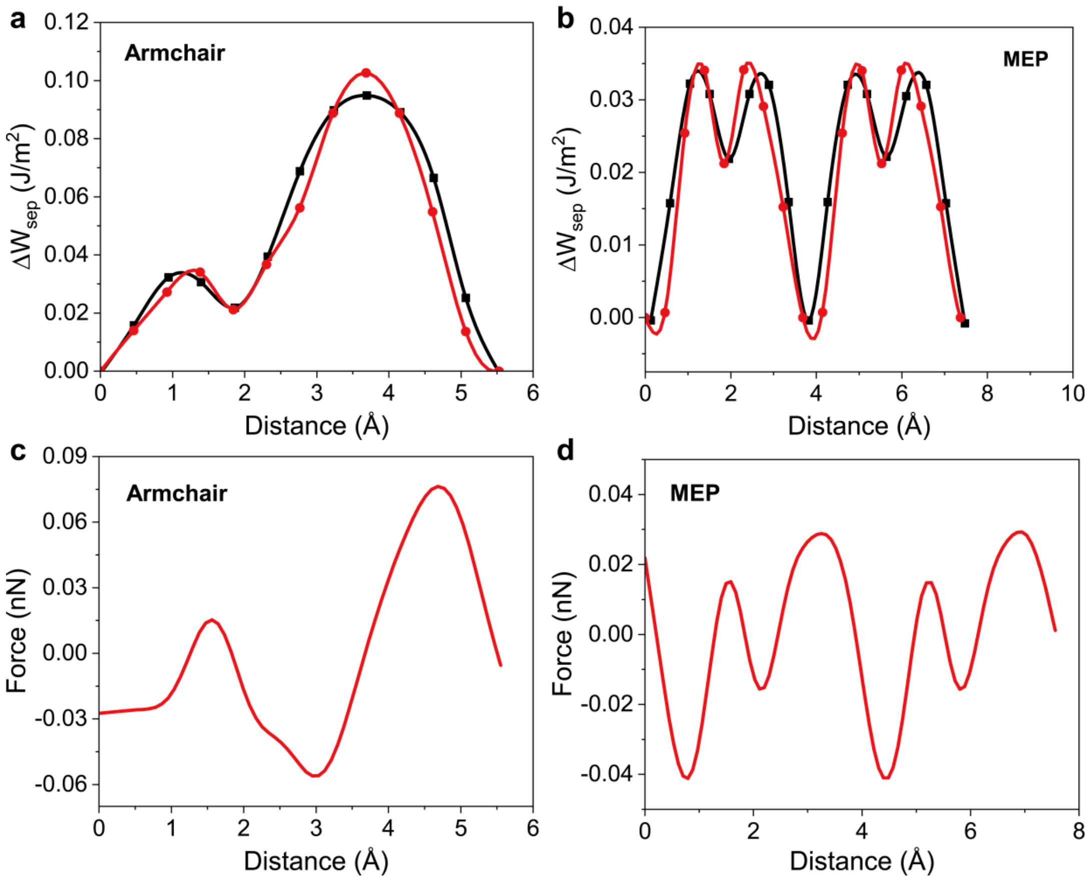

Supplementary Fig. 12 Work of separation and friction force during sliding. (**a, b**) Variation of work of separation with the sliding distance along the armchair direction and MEP. The red lines are from our DFT calculations, while the black lines are from previous DFT calculations[15]. (**c, d**) Variation of friction force with the sliding distance along the armchair direction and MEP.

7. Validation of our theoretical model and comparison with previous fixed-boundary model

Our theoretical model considers a finite interfacial shear stress between the tested membrane and the substrate. Thus, our model is called as the shearing-boundary model. When the interlayer shear stress τ tends to infinity in Eq. (2), our shearing-boundary model can be degraded into previous fixed-boundary model (i.e. Eq. (1) in main text).

It indicates that the fixed-boundary model is only a limit case of our shearing-boundary model. Therefore, our shearing-boundary model is more general compared with the fixed-boundary model.

To further validate our shearing-boundary model, we used our shearing-boundary model to fit the nanoindentation force-displacement curves of graphene on SiO_2 reported in the literature[3]. The obtained values of shear stress τ, pretension σ_0, and modulus E for 1.5-μm-diameter hole nanoindentation are 3.2 MPa, 810.6 MPa, and 1127.3 GPa, respectively. The obtained values of τ, σ_0, and E for 1-μm-diameter hole nanoindentation are 6.8 MPa, 1061.4 MPa, and 1183.6 GPa, respectively. These values of σ_0 and E are close to those reported in the literature[3]. Especially, the value of shear stress τ for graphene on SiO_2 substrate from 1.5-μm-diameter hole nanoindentation is close to that (1-3 MPa) measured by pressurized microscale bubbling[4]. These results indicate the validity of our shearing-boundary model and also clarify its advance compared to previous fixed-boundary model.

Supplementary Table 1 Parameters used in 12-6 LJ potentials of systems including Mo, S, Si, and O.

System	ε (eV)	σ (nm)
Mo-Si	0.00320	0.401
S-Si	0.01867	0.348
Mo-O	0.00123	0.366
S-O	0.00721	0.347
Mo-S	0.00456	0.317
Mo-Mo	0.00094	0.360
S-S	0.02218	0.325

Supplementary References

1 Wan, K. T., Guo, S. & Dillard, D. A. A theoretical and numerical study of a thin clamped circular film under an external load in the presence of a tensile residual stress. *Thin Solid Films* **425**, 150-162, (2003). doi: 10.1016/S0040-6090(02)01103-3.

2 Komaragiri, U. & Begley, M. R. The mechanical response of freestanding circular elastic films under point and pressure loads. *J. Appl. Mech-T Asme.* **72**, 203-212, (2005). doi:10.1115/1.1827246.

3 Lee, C., Wei, X., Kysar, J. W. & Hone, J. Measurement of the elastic properties and intrinsic strength of monolayer graphene. *Science* **321**, 385-388, (2008). doi:10.1126/science.1157996.

4 Wang, G. *et al.* Measuring interlayer shear stress in bilayer graphene. *Phys. Rev. Lett.* **119**, 036101, (2017). doi:10.1103/PhysRevLett.119.036101.

5 Plimpton, S. Fast parallel algorithms for short-range molecular-dynamics. *J. Comput. Phys.* **117**, 1-19, (1995). doi:DOI 10.1006/jcph.1995.1039.

6 Ding, B. *et al.* Brittle versus ductile fracture mechanism transition in amorphous lithiated silicon: From intrinsic nanoscale cavitation to shear banding. *Nano Energy* **18**, 89-96, (2015). doi:10.1016/j.nanoen.2015.10.002.

7 Liang, T., Phillpot, S. R. & Sinnott, S. B. Parametrization of a reactive many-body potential for Mo-S systems. *Phys. Rev. B* **79**, 245110, (2009). doi:10.1103/PhysRevB.79.245110.

8 Munetoh, S., Motooka, T., Moriguchi, K. & Shintani, A. Interatomic potential for Si-O systems using Tersoff parameterization. *Comput. Mater. Sci.* **39**, 334-339, (2007). doi:10.1016/j.commatsci.2006.06.010.

9 Gabourie, A. J., Suryavanshi, S. V., Farimani, A. B. & Pop, E. Reduced thermal conductivity of supported and encased monolayer and bilayer MoS_2. *2D Materials* **8**, 011001, (2020). doi: 10.1088/2053-1583/aba4ed.

10 Jung, G. S., Wang, S., Qin, Z., Martin-Martinez, F. J., Warner, J. H. & Buehler, M. J. Interlocking friction governs the mechanical fracture of bilayer MoS_2. *ACS Nano* **12**, 3600-3608, (2018). doi: 10.1021/acsnano.8b00712.

11 Rozman, M. G., Urbakh, M. & Klafter, J. Stick-Slip Motion and Force Fluctuations in a Driven Two-Wave Potential. *Phys. Rev. Lett.* **77**, 683-686, (1996). doi:10.1103/PhysRevLett.77.683.

12 Kresse, G. & Furthmüller, J. Efficient iterative schemes for ab initio total-energy calculations using a plane-wave basis set. *Phys. Rev. B: Condens. Matter.* 54, 11169 (1996). doi: 10.1103/PhysRevB.54.11169.

13 Perdew, J., Burke, K. A. & Ernzerhof, M. Generalized gradient approximation made simple. *Phys. Rev. Lett.* **77**, 3865 (1996). doi: 10.1103/PhysRevLett.77.3865.

14 Grimme, S. Semiempirical GGA-type density functional constructed with a long-range dispersion correction. *J. Comput. Chem.* **27**, 1787 (2006). doi: 10.1002/jcc.20495.

15 Levita, G., Molinari, E., Polcar, T. & Righi, M. C. First-principles comparative study on the interlayer adhesion and shear strength of transition-metal dichalcogenides and graphene. *Phys. Rev. B* **92**, 085434 (2015). doi: 10.1103/PhysRevB.92.085434.
